# Medical Opinion and Movement

**Published:** 1908-06-13

**Authors:** 


### Medical Opinion and Movement.

At a recent meeting of the Medical Society of Vienna, Dr. Schick communicated the results of his experiments with diphtheria toxin, to find if it could be used for the diagnosis of diphtheria by subcutaneous and ophthalmic methods, in the same way as von Pirquet and Calmette-Eisner have used tuberculin. Pure toxin proved entirely negative, but Schick was able to produce a transient reaction, in many ways similar to the von Pirquet tuberculin reaction, by using a 10 per cent. solution of diphtheria toxin. He regards this reaction as being specific. The best results are obtained in children between three and seven years of age; after ten years the reaction is a very feeble one.

One of the most interesting cases demonstrated before the recent German Surgical Congress, was a woman of thirty, who had been operated upon by Professor Hochenegg, of Vienna, for a hypophyseal tumour, causing acromegaly (?), and symptoms of intra-cranial pressure. The patient, who up to her twenty-fifth year had been normal and in good health, had suffered from anæmia, headache, and lassitude since 1902. Four years ago typical acromegalic symptoms were first noticed, which up to the time of operation were getting steadily worse. Owing to the intensely painful neuralgic pains and the constant headache, it was decided to operate. The hypophysis was reached through the anterior route, and a tumour, the size of a small walnut, which proved on examination to be an adenoma, was removed. The patient made a quick and uninterrupted recovery, and was able to leave her bed ten days after the operation. Still more satisfactory was the sudden decrease in the acromegaly which was noticed as early as the fourth day after the operation. Patient's hands and feet decreased in size, and a diminution could also be proved, by measurement, to have taken place in the facial bones. The mental condition was much improved, the woman being brighter and taking more interest in her environment than when she entered the ward, and her condition at present may be looked upon as steadily improving.

In discussing this case, Hochenegg concluded that a tumour of the hypophysis or pituitary body is the cause of acromegaly, and that it is the main factor in producing the characteristic facial changes, while the peripheral changes—in the hands and feet—are probably occasioned, or influenced, by spinal conditions. The operation is of interest, as showing that the removal of such a tumour may not only ease the local symptoms dependent upon the pressure exercised by the growth, but that it may likewise benefit the general condition of the patient in a marvellous degree. At the same meeting, Professor Borchard, of Berlin, showed a case in which he had partially removed a similar growth of the hypophysis. The operation was satisfactory, in so far that the severe headaches no longer troubled the patient, though her general condition was not much bettered. The president of the Congress, Professor Eiselsberg, stated that he had thrice removed a pituitary tumour. The first case was a malignant growth, and the operation only temporarily benefited the patient; the second was a case of acromegaly, due to sarcoma, and the patient died of meningitis, while the third tumour was also malignant.

Fibrolysin has been at the service of the profession now for some time, but does not appear to have accumulated much faith in its efficacy, at any rate in this country. Whether this is because it has not received a fair trial or because it has been tried and found wanting, it is difficult to say. From time to time, however, reports continue to appear in Continental medical literature in support of its therapeutic claims. As the name implies, it is supposed to exercise a special influence upon fibrous scar tissue, and to cause it to soften and to disappear. It is a chemical combination of thiosinamine and sodium salicylate. Thiosinamine was originally introduced by Hebra in 1892 for the treatment of lupus. Clinically it is allyl-sulpho-urea, and it was found that this preparation has the power to soften scar tissue. Fibrolysin is soluble in water, and is stable when kept from air and light. It may be administered by intravenous or intramuscular injection in doses of 2-3 cubic centimetres, and the injections may be given every one, two or three days. Some good results have been reported by Becker in cases of Dupuytren's contraction, and in cases of stiff joints. Lang has reported recently on successful results he has obtained in cases of urethral stricture. The injections are occasionally followed by transient constitutional symptoms—headache, sleepiness and general malaise. According to F. Mendel, satisfactory results cannot be looked for unless active dilatation of the scar tissue can be carried out at the same time. The action of the drug is to soften the tissue, and so assist its dilatation.

Nystagmus has been associated by physiologists for some time with changes in the semicircular canals, and clinicians have observed the phenomenon in connection with manifestations of vertigo, but the subject has only been thoroughly worked out recently by Barany, of Vienna. According to this authority, the reflex nystagmus of the eyeballs set up by excitation of the vestibular part of the auditory apparatus affords an important diagnostic sign in morbid conditions of the ear. Ewald, experimenting on pigeons, showed that movement of the endolymph in a canal towards the ampulla caused a movement of the head in the same plane and in the same direction. Movement of the fluid away from the ampulla caused movement of the head in the opposite direction. In man this movement of the head is replaced by a movement of the eyeballs, and constitutes the reflex nystagmus which is under consideration. This reflex nystagmus is rythmic in character, and consists of two movements —a slow movement and then a rapid one in the opposite direction. The direction of the nystagmus is determined by the direction of the rapid movement.

A spontaneous nystagmus may be set up by an acute affection of the labyrinth, associated with vertigo. In normal individuals the reflex nystagmus can be excited in several ways: by rotation, by thermal changes, by the galvanic current, and by compression of air in the auditory meatus.

---

THE thermal method is the simplest. The apparatus for the purpose is simple and the patient can be examined by it in bed. Hot or cold water is injected into the meatus through a fine cannula, without exciting any pressure beyond what is necessary to establish a current. With the head upright, cold water injected into the right ear produces a rotatory nystagmus to the left. Injection of hot water results in a rotatory nystagmus to the right. Barany explains these phenomena by the fact that with the head upright the arc of the anterior vertical canal forms the highest point of the vestibular apparatus, and its ampulla is nearest to be affected by the hot or cold water. The change of temperature causes movements in the endolymph by changes in the density of the fluid and corresponding ocular movements as in the case of rotation of the head. If the head is inclined to the left shoulder the arc of the horizontal canal then becomes the highest point, and the endolymph of this canal is especially affected by the injections. Consequently cold water injected into the right ear with the head inclined to the left results in a horizontal nystagmus to the right, an injection of hot water causes a nystagmus to the left in the same plane. The application of this reflex to clinical purposes consists in the determination of its presence or absence or of variations from the normal. MM. Lombard and Halphen, discussing the subject in *Le Progrès Medical*, report a number of cases in which examination for the reflex has proved of service in diagnosis. Absence of the reflex is evidence of a condition of so-called *alabyrinthie vestibulaire.* It is frequently absent in cases of tabes dorsalis. On the other hand, in cases of vertigo, if the reflex is normal the cause for the vertigo must be looked for elsewhere than in the terminal fibres of the vestibular nerve. Exaggeration of the reflex on one side especially in point of duration indicates a condition of hypersensitiveness due to vestibular inflammation of that side. The phenomena is an interesting one, and will doubtless find its place in the clinical investigation of nervous diseases.

---

THE successful results which have followed the injection of alcohol in cases of neuralgia of the trigeminal nerve and of tic douloureux of the face have naturally led to the application of the same remedy to neuralgias of other nerves of the body, and in particular to cases of sciatica. With certain authorities this method of treatment, in cases of sciatica, for instance, has been followed by equally satisfactory results; Schlösser reports on 35 cases of obstinate sciatica, in 36 of which he was able to effect a cure. On the other hand, a more extended use of the method has shown that it is fraught with considerable danger when applied to a motor or mixed nerve. The injections not infrequently are followed by an acute neuritis resulting in more or less paralysis of the nerve and muscles supplied by it. The variable results which have been obtained appear to depend largely upon whether the injection is made directly into the nerve trunk or into the adjacent tissue and the effect also probably varies according to the original condition of the nerve. Dr. Felix Allard has given the subject considerable attention and reports on several cases which have been treated by this method and which he has subsequently examined electrically. He finds that the injections of alcohol set up a condition of neuritis more or less intense, which is evidenced by the usual electrical reactions. He emphatically condemns the use of the method to motor or mixed nerves owing to the impossibility of determining the exact conditions of the injection in relation to the nerve and the amount of inflammatory reaction which a particular injection may set up. He thinks, however, it may be usefully employed in certain cases of spasm and in the contractures of hemiplegics and paraplegics.

---

THE ætiology of diabetes is still one of the most fascinating of the many puzzles which claim the attention of the physician and surgeon alike. Notwithstanding the large amount of research that has been done on the subject in its various aspects, it cannot be truthfully said that we are much nearer a solution of the problems concerning the origin of this disease than we were before Von Mering and Minskowski made the interesting discovery that some forms of diabetes are intimately associated with lesions of the pancreas, and that extirpation of this organ in animals promptly leads to glycosuria in an exaggerated degree. Recently, however, new light has been shed on the subject by the researches of certain pathologists, who found that it was practically impossible to produce glycosuria in animals whose thyroids had been removed. At the recent Medical Congress in Vienna a paper was read on the subject in which the authors communicated the results of an extended series of observations. It was found that in dogs whose thyroids had been completely removed it is not possible to produce glycosuria by pancreatic extirpation or by phloridzin injections. In rabbits the results are equally interesting, for here even puncture of Claude Bernard's point at the calamus failed to produce diabetes. These findings open up a wide field for further research and investigation, no less interesting from a clinical than from the purely physiological and pathological point of view. The authors propose to carry on their researches, and promise a full and extended report on their collected observations at an early date.

---

THROUGH the death of Professor Hoffa the University of Berlin lost its special professor of orthopædic surgery, and there seems to be some difficulty in filling the chair. The late Professor Hoffa came to Berlin on a special invitation, and the chair of orthopædic surgery was specially created for him, while he was promised certain privileges which were not shared by other "special" professors of the faculty. Notwithstanding the great success of his work and the fact that his connection with the Berlin University was a standing advertisement that drew hundreds of medical students to it, these promises were not fully carried out. Thus no beds were assigned to him, and he had to carry on his work in confined quarters and under many disadvantages. When the chair became vacant through his death there was some fear that the authorities would abolish it altogether. Against this alleged danger the Orthopædic Congress protested vigorously, and the protest elicited from the authorities the promise that every endeavour would be made to fill the vacancy as soon as possible. Nor has there been much delay. The first to be called was Professor Lange, of Munich, whose work in tendon transplanting is so well known. We now learn that Professor Lange has declined the call, mainly because the authorities have refused to grant him more than half a dozen beds. The next on the list is said to be Professor Klapp, who is the chief assistant to Professor Bier, but it is doubtful whether the authorities can overlook the claims of Professor Joachimsthal, who is, perhaps, the best-known orthopædist in Germany, and whose work is universally recognised and appreciated.

---

In any case, no good man will undertake the work and its by no means light responsibilities unless adequate provision is made to enable him to cope with the mass of material that confronts the orthopædic surgeon in Berlin. Hitherto this branch of surgery has been the Cinderella of specialities. The general surgeon has thought himself quite able to deal with orthopædic cases, and has set his face against separating this branch of work from general surgery. Where a special chair has been created for the orthopædic lecturer he has had to do his work under most unfavourable conditions and very often severely handicapped. Thus Professor Lorenz at Vienna works in a tiny and utterly inadequate out-patient department, and has no beds of his own. Moreover, as he pointed out, with smiling bitterness, at the recent congress, the authorities are only waiting for his death to abolish the chair altogether. Nor is the orthopædist treated better with us. In some cases the special work in this very important branch is assigned to the junior assistant surgeon, who has no particular qualification for it, and who treats it as part of his general work. In other hospitals no orthopædic department exists. We hold no brief for the specialist, and we do not favour an indiscriminate multiplication of separate departments in hospital work; but orthopædy has firmly established itself, and has proved itself worthy of being considered as much a speciality as eyes, ears, or throats. The surgeon who is unacquainted with its niceties and knows it only in a general way will do as little good by dabbling in it as the man who undertakes expert eye work without a knowledge of "refractions." Those who are interested in the advancement of surgery generally will look with keen concern towards the Berlin University in its efforts to find a successor to Hoffa, and will cordially endorse its decision not to abolish the chair which its first occupant had filled so brilliantly.

The treatment of painful corns and callosities of the foot is difficult, slow, and often unsatisfactory, so that a communication of Peugniez to *l'Electricité Médicale*, in which he calls attention to the successful result of the application of *x*-rays to the feet in such conditions, is worthy of note. The author had himself suffered for five months from plantar callosities, which had caused him such intense pain and impairment of function that he was unable to take any form of exercise. Every remedy in turn was tried, but without relief resulting therefrom. At last the services of a skilled radiographer were called in, and relief at once resulted from the first application of the rays. Six applications, made successively at intervals of a week, completed the cure. Peugniez does not claim the treatment as original, for Guillemonat, of Lyon, first drew attention to the use of *x*-rays in such conditions of the feet in 1906. He merely claims that the treatment is simple in application, inexpensive and efficient, if carried out by a skilled operator. The rate of improvement under small doses of the rays is astonishing. We can see no objection to his idea, providing a suitable operator can be found to administer the rays. Otherwise it is better to rely on salicylic acid and kindred remedies, for burns due to the unskilful application of *x*-rays would but add new anguish to an already extremely painful condition.

---

Lourties, in the *Journal des Practiciens*, publishes an account of some 20 cases of pulmonary tuberculosis, treated by him with injections of biliary extracts, after the method introduced by Professors Lemoine and Gérard of Lille, based on the antitoxic action of the liver. The series of cases comprised all stages of the disease, with the usual symptoms and signs, cough, fever, night sweats, bacilli in the sputum, etc.; and marked improvement is said to have taken place in all cases save two, the distressing symptoms gradually disappearing, and the patients rapidly gaining in weight. Moreover, in cases in which from one reason or another treatment had been stopped, rapid deterioration set in, and symptoms reappeared. The treatment consists in the injection, sub-cutem, of 2 c.c. of paratoxin every second day, continued over a period of several months, sufficiently long to exclude the possibility of the amelioration being merely due to change of treatment: a phenomenon by no means rarely seen by those who deal with patients of such a sanguine temperament as the tuberculous. Lourties hazards the opinion that it is possible that the benefit often derived from taking cod-liver oil in this disease is to be explained by the specific action of the biliary extracts contained in the oil. He similarly explains the undoubted immunity from tuberculosis possessed bp people who are subject to joint affections, as being the result of the marked hypersecretion of bile found in such persons. He is led to think that the action of the biliary extracts is specific from the fact that doubtful cases of the disease in which no bacilli could be found in the sputum, derived no benefit from treatment. The treatment, he maintains, is rational, since it is well-known that cholesterine has an antitoxic action on the venom of the cobra; moreover Almaggia has recently reported two cases of tetanus, cured by injection of the same drug.

